# PAK4 Regulates Actin and Microtubule Dynamics during Meiotic Maturation in Mouse Oocyte

**DOI:** 10.7150/ijbs.34718

**Published:** 2019-09-07

**Authors:** Ya-Ting He, Lei-Lei Yang, Shi-Ming Luo, Wei Shen, Shen Yin, Qing-Yuan Sun

**Affiliations:** 1 College of Animal Science and Technology, College of Life Sciences, Institute of Reproductive Science, Key Laboratory of Animal Reproduction and Germplasm Enhancement in Universities of Shandong, Qingdao Agricultural University, Qingdao 266109, China;; 2 State Key Laboratory of Stem Cell and Reproductive Biology, Institute of Zoology, Chinese Academy of Sciences, Beijing 100101, China.

**Keywords:** oocyte, meiosis, PAK4, cytoskeleton, centrosome.

## Abstract

Meiotic maturation of oocyte is an important process for successful fertilization, in which cytoskeletal integrality takes a significant role. The p-21 activated kinases (PAKs) belong to serine/threonine kinases that affect wide range of processes that are crucial for cell motility, survival, cell cycle, and proliferation. In this study, we used a highly selective inhibitor of PAK4, PF-3758309, to investigate the functions of PAK4 during meiotic maturation of mouse oocytes. We found that PAK4 inhibition resulted in meiotic arrest by inducing abnormal microfilament and microtubule dynamics. PAK4 inhibition impaired the microtubule stability and led to the defective kinetochore-microtubule (K-M) attachment which inevitably resulted in aneuploidy. Also, PAK4 inhibition induced abnormal acentriolar centrosome assembly during meiotic maturation. In conclusion, all these combined results suggest that PAK4 is necessary for the oocyte meiosis maturation as a regulator of cytoskeleton.

## Introduction

Meiosis is characterized by two continuous rounds of cell divisions accompanied with only one round of DNA replication, which is highly associated with the maturity and formation of haploid gametes during sexual reproduction 1-3. Oocyte meiosis is a highly asymmetric cell split, and eventually produces a mature egg for fertilization, in which cytoskeletal integrality plays important roles 4, 5.

From birth until ovulation in puberty, female mammalian oocytes are persistently arrested in the early stage of meiosis I, in which there is a distinct germinal vesicle (GV) inside. Before ovulation, oocytes resume the meiotic maturation from GV stage and finish the first polar body extrusion (PBE) to reach the metaphase of meiosis II (MII) waiting for binding sperm to be fertilized. This meiotic maturation procession is very important and contains a series of complex and multiple steps for nuclear maturation and cytoplasmic maturation, including germinal vehicle breakdown (GVBD), faithful chromosome alignment and segregation, cytoskeletal integrality, etc. 6, 7. Cytoskeleton refers to the well-organized and complex structure of fiber networks in eukaryotic cells. It plays many important roles in maintaining cell morphology and bearing external stress like skeleton in human body, and participates in many important cellular activities 8. Cytoskeleton consists of intermediate filament fibers, microfilaments and microtubules 8. Intermediate filaments do not undergo clear changes during meiotic maturation of oocytes, so main concerns about cytoskeleton are focused on microfilaments and microtubules 4. As a cytoskeleton component, microfilaments are involved in various events in meiotic maturation and fertilization of oocytes, including GVBD, meiotic spindle formation, faithful chromosome alignment and segregation, PBE, etc. 9. Microtubules are usually classified according to the positional connection, such as kinetochore microtubules, star microtubules, and microtubules connected to the chromosome arms 10. In meiosis I, the kinetochore of homologous chromosome is connected to the kinetochore microtubules from the opposite poles of spindle to ensure the correct homologous chromosome bi-orientation 11-13. Abnormal kinetochore-microtubule (K-M) attachment leads to unfaithful homologous chromosome segregation, which finally causes aneuploidy in subsequent maturation. Also, during meiosis I in oocyte, acentriolar centrosome remodeling plays essential roles for the correct formation of meiotic spindle, specifically to separate homologous chromosomes accurately. Centrosome abnormality inevitably leads to the inaccurate microtubule organization dynamics and abnormal chromosome alignment, with unfaithful chromosome segregation resulting in the aneuploidy 4.

The p21-activated kinases (PAKs) are a class of conserved serine/threonine protein kinases which participate in many biological functions through binding some downstream proteins and kinase substrates, specially considered to be associated with cancer cells 14-18. In mammals, two groups of PAKs have been identified based on the sequence homology, in which group I (PAK 1-3) has an inhibitory region, while group II (PAK 4-6) does not 19. PAK4 is the earliest discovered and the most representative member in PAKs group II 20. Up to now, it has been reported that PAK4 plays important roles for efficient mitosis progression, such as PAK4 regulates the membrane foaming, spindle localization and the G1 phase and G2/M phase 21, 22. Also, PAK4 inhibition is associated with oncogenic transformation by protecting apoptosis 23, inhibiting cell adhesion 24 and promoting cell migration 25. Although PAK4 is the earliest discovered and the most representative member in PAKs group II, its functions during meiosis still remain unknown.

One highly selective inhibitor of PAK4, PF-3758309, has been reported to be widely used in cancer cell research 14, 15, 18. In this study, we aimed to investigate the functions of PAK4 in cytoskeleton regulation by its inhibitor PF-3758309 during meiotic maturation of mouse oocytes. Our data indicated that PAK4 inhibition may cause meiotic arrest by inducing abnormal actin and microtubule dynamics during meiotic progression. PAK4 inhibition leads to the abnormal K-M attachment and subsequent aneuploidy. Also, PAK4 inhibition induces the abnormal acentriolar centrosome assembly. Therefore, all these results suggest that PAK4 is necessary for oocyte meiosis as a regulator of the cytoskeleton.

## Materials and methods

### Animals and ethics statement

All the experiments were performed consistent to the guidelines of Animal Research Institute Committee of Qingdao Agricultural University. With appropriate dark/light cycles, four-week-old female ICR mice were raised and fed with normal diet in a temperature-controlled room. All the mice were performed quickly and humanely during sample collection.

### Oocyte culture

Only germinal vesicle (GV) intact oocytes were selected in M2 medium and cultured in M16 medium (Sigma-Aldrich, St. Louis, MO, USA) under paraffin oil at 37℃ in a 5 % CO_2_ atmosphere for maturation *in vitro*. Oocytes were collected at different time points after culture for further analysis. PF-3758309 (MCE, Shanghai, China), as a selective potent PAK4 inhibitor, was dissolved in DMSO at a 10 mM stock solution 15. The final working concentration is 0, 1, 10 or 50 μM (DMSO concentration is less than 0.3%). According to the rate of PBE, the subsequent experiments were performed with the concentration of 1 μM, unless specified.

### Nocodazole and paclitaxel treatment

The GV intact oocytes were cultured in M16 medium for 8 h to metaphase of meiosis I (MI). Then, oocytes were treated with 5 μg/mL nocodazole (Sigma-Aldrich, St. Louis, MO, USA) for 5 min, accompanied with or without 1 μM PF-3758309. Or, the GV intact oocytes were cultured for 2 h to GVBD, and then cultured with 0.04 μg/mL Nocodazole for 6 h to MI, accompanied with or without 1 μM PF-3758309 26, 27. For paclitaxel (MCE, Shanghai, China) treatment, the GV intact oocytes were cultured in M16 medium with 0.5 μM paclitaxel for 8 h to MI, accompanied with or without 1 μM PF-3758309 as previously described 26.

### Immunofluorescence

Oocytes were fixed in 4% paraformaldehyde (PFA) for 30 min and permeabilized for 20 min at room temperature (RT) in PBS containing 0.5% Triton X-100. After blocking for 1 h in PBS containing 1% bovine serum albumin (BSA), oocytes were incubated with different primary antibodies overnight at 4 ℃. Next day after washing, oocytes were incubated with a suitable secondary antibody for 2 h at RT. For staining DNA, DAPI (Beyotime Biotechnology Institute, Shanghai, China) was used for 15 minutes or PI (Sangon Biotech, Shanghai, China) was used for 10 minutes. Finally, oocytes were mounted on glass slides with DABCO droplets. Images were captured under the same settings by a laser scanning confocal microscopy (Leica TCS SP5 II, Wentzler, Germany). At least three replicates were performed with more than 20 oocytes observed per group. Primary antibodies include rabbit anti-PAK4 antibody (1:50, Sangon Biotech, Shanghai, China), rabbit anti-tubulin antibody (1:100, Sangon Biotech, Shanghai, China), mouse monoclonal anti-tubulin antibody (1:250, Sangon Biotech, Shanghai, China), TRITC-conjugated Phalloidin for actin (1:1000, AAT Bioquest, Sunnyvale, CA, USA), Rabbit anti-Pericentrin (centrosome marker) antibody (1:50, Abcam, Cambridge, UK), Human anti-Centromere protein antibody (1:200, Antibodies Incorporated, Davis, CA, USA). Secondary antibodies include Cy3-labeled Goat Anti-Rabbit IgG (H+L) (1:200, Beyotime Biotechnology Institute, Shanghai, China), FITC-labeled Goat Anti-Rabbit IgG (H+L) (1:200, Beyotime Biotechnology Institute, Shanghai, China), FITC-labeled Goat Anti-Mouse IgG (H+L) (1:200, Beyotime Biotechnology Institute, Shanghai, China), Cy3-conjugated Donkey Anti-Human IgG (1:200, Sangon Biotech, Shanghai, China).

### RNA extraction and quantitative real-time PCR (qPCR)

Total RNA was prepared from oocytes by using an EZ-10 Spin Column Total RNA Isolation Kit (Sangon Biotech, Shanghai, China). HiScript II Q RT SuperMix (Vazyme Biotech Co. Ltd, Nanjing, China) was used for the first cDNA strand. AceQ qPCR SYBR Green Master Mix (Vazyme Biotech Co. Ltd, Nanjing, China) was used for Quantitative real-time PCR (qPCR) in Applied Biosystems 7500 Sequence Detection System. The final 20 μL reaction volume was 2 μL of cDNA (100 ng), 10 μL of SYBR Green Master Mix, 0.4 μL of ROX Reference Dye 2, 0.4 μL of each primer (10 μM) and 7.2 μL of RNase free H_2_O. PCR amplification conditions were as follows: reaction was initiated at 95℃ for 10 min, followed by 40 sequential cycles of denaturation (15 s at 95℃), annealing (30 s at 60℃) and extension (20 s at 72℃). Triplicate samples for *PAK4* gene were evaluated. The threshold cycle values were calculated with mean ± SD. The primers are F-TCTGACCAGCGGGACAAAAC and R- GCTGAAGGCCCATTAGGGG.

### Chromosome spread

Oocytes were put into acid M2 for 5 seconds to remove the zona pellucida. After washing, zona pellucida-free oocytes were fixed on a slide with a drop of 1% PFA, 0.15% Triton X-100 and 3 mM dithiothreitol (DTT, Sangon Biotech, Shanghai, China). After drying, the chromosomes were stained with PI and examined under a laser scanning confocal microscope 28.

### Statistical analysis

All experiments were repeated at least three times. Results were expressed as mean ± SEM and analyzed by one-way ANOVA analysis with SPSS software (IBM Co., USA).* P*-value <0.05 was considered to be a statistically significant difference.

## Results

### PAK4 inhibition impairs the normal meiosis progression in mouse oocyte

**Figure 1:**
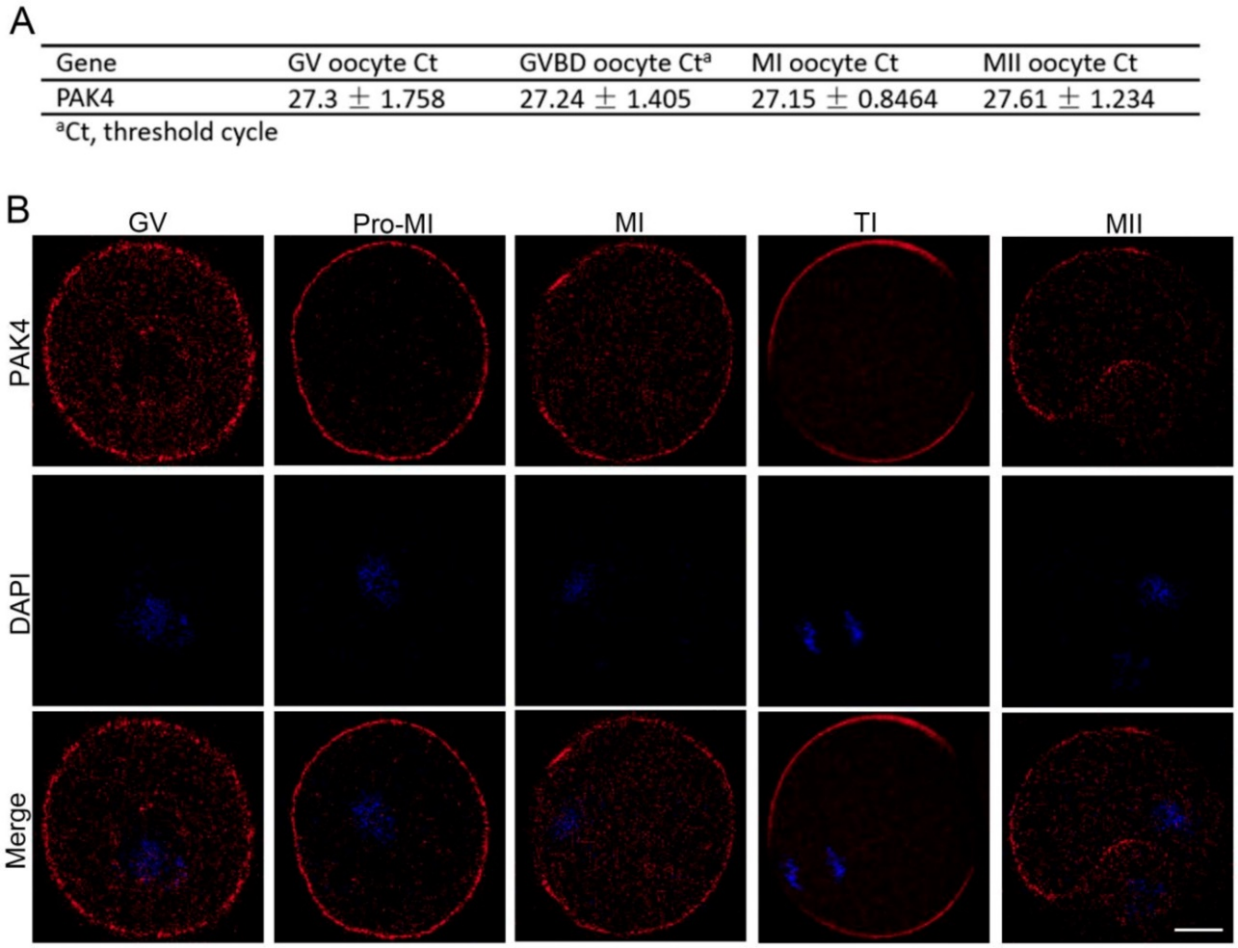
** PAK4 expression and subcellular localization during meiotic maturation of mouse oocytes. (A)** Expression levels of PAK4 mRNA at different development stages after oocyte meiosis resumption. The GV (germinal vehicle) stage, 0 h; GVBD (germinal vehicle breakdown) stage, 2 h; MI (metaphase I) stage, 8 h; MII (metaphase II) stage, 12 h. **(B)** Immunofluorescent staining for PAK4 (*red*) and DNA (*blue*) at the GV, Pre-MI (pre-metaphase I), MI, TI (telophase I) and MII stages in mouse oocytes. Bar = 20 μm.

First of all, we need to prove that PAK4 is present during meiotic maturation in mouse oocyte. The mRNA expression of *PAK4* was detected by qPCR corresponding to different development stages, including the GV, GVBD, MI, MII stages, during meiotic maturation (Figure 1A). Immunofluorescent staining showed that PAK4 mainly localized in the plasma membrane and cytoplasm during oocyte meiosis maturation (Figure 1B), consistent to the description of previous report in mitosis 29. These results revealed that PAK4 is present throughout various stages of meiotic maturation in mouse oocyte.

**Figure 2:**
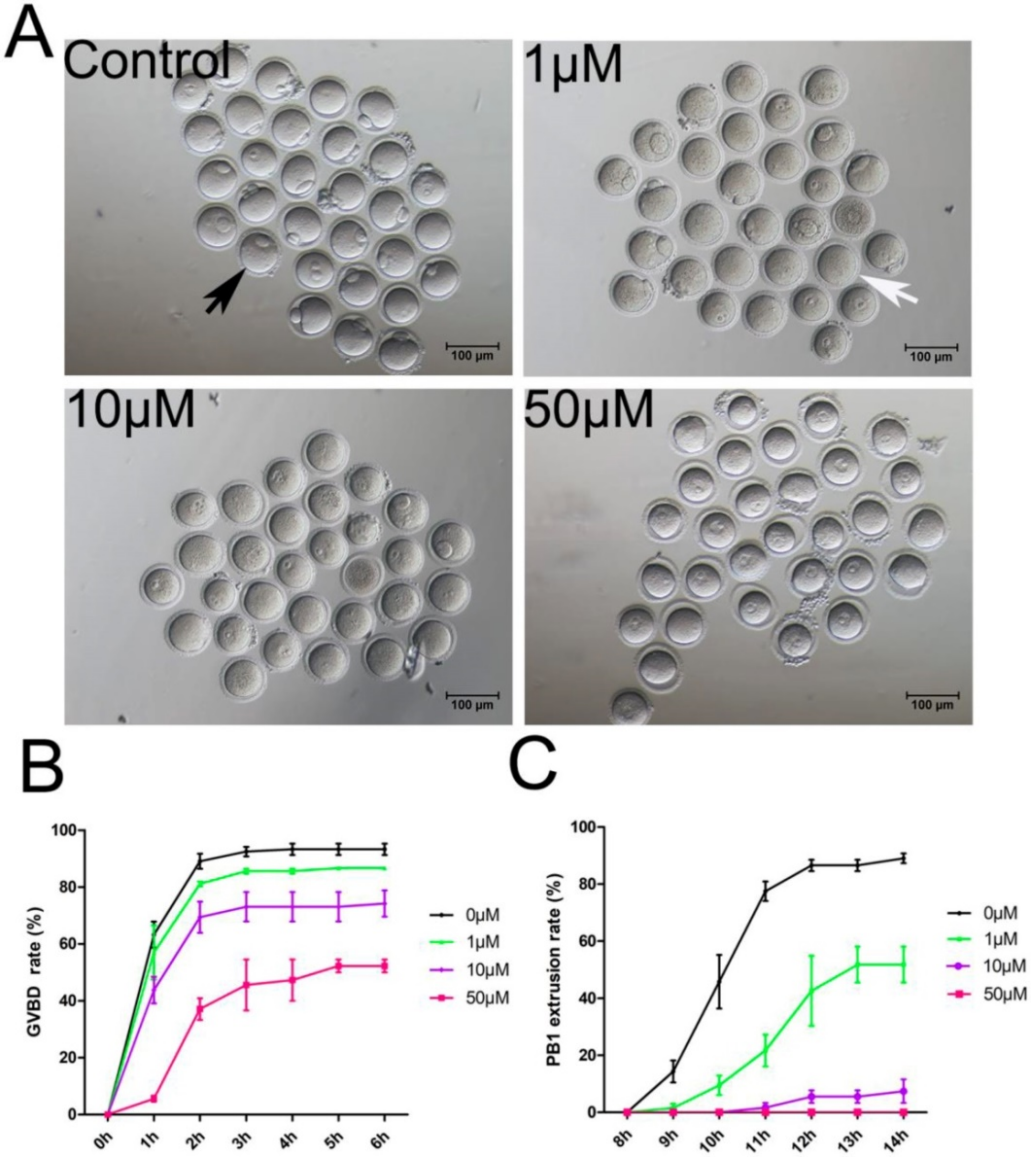
** PAK4 Inhibition impairs the meiotic maturation in mouse oocytes. (A)** Representative images of oocyte maturation cultured for 12 h with increasing concentrations of PF-3758309 (PAK4 inhibitor). Black arrow indicates oocyte with the first polar body, while white arrow indicates oocyte without the first polar body. Bar = 100 μm. **(B)** Quantitative analysis of GVBD rates after 2 h culture in the control, 1, 10 and 50 μM PF-3758309 treatment groups. **(C)** Quantitative analysis of the first polar body extrusion (PBE) rates after 12 h culture in the control, 1, 10 and 50 μM PF-3758309 treatment groups. Data are presented as mean ± SEM of at least three independent experiments.

To study the PAK4 functions, we inhibited PAK4 functions by a highly selective potent inhibitor of PAK4, PF-3758309, which is widely used in mitosis before 14, 15, 30. Oocytes were cultured for *in vitro* maturation with different concentrations of PF-3758309 at 0 (control), 1, 10 or 50 μM (Figure 2A). The GVBD rate of oocytes was continuously observed under stereoscopic microscope from 1h to 6h of culture as reported before 31. The results showed that the GVBD rate decreased significantly in the PF-3758309-treated group (Figure 2B). After culture for 2 hours, the GVBD rates in the control, 1, 10 and 50 μM PF-3758309 treatment groups are 89.08 ± 2.64%, n = 120 vs 81.14 ± 0.83%, n = 90, P > 0.05; vs 69.40 ± 5.50%, n = 86, P < 0.01; vs 37.12 ± 3.79%, n = 62, P < 0.01. The PBE rate of oocytes was continuously observed under stereoscopic microscope from 8h to 14h of culture as reported before 31. Consistently, the rate of PBE decreased significantly in the PF-3758309-treated group (Figure 2C). At 12 hours of culture, the PBE rates in the control, 1, 10 and 50 μM PF-3758309 treatment groups are 86.52 ± 2.01%, n = 119 vs 42.57 ± 12.27%, n = 64, P < 0.01; vs 5.51 ± 2.18%, n = 56, P < 0.01; vs 0 ± 0%, n = 60,* P <* 0.01. All these results indicate that PAK4 is essential for meiotic maturation progression in mouse oocyte. As higher concentration of PF-3758309 can lead to the unexpected inhibition, people set up a gradient concentration and chose the small one to perform their experiments 15, 30. Based on the results, we selected 1 μM PF-3758309 as the experimental group concentration for subsequent tests.

### PAK4 inhibition results in abnormal actin and microtubule dynamics during meiotic maturation in mouse oocyte

**Figure 3:**
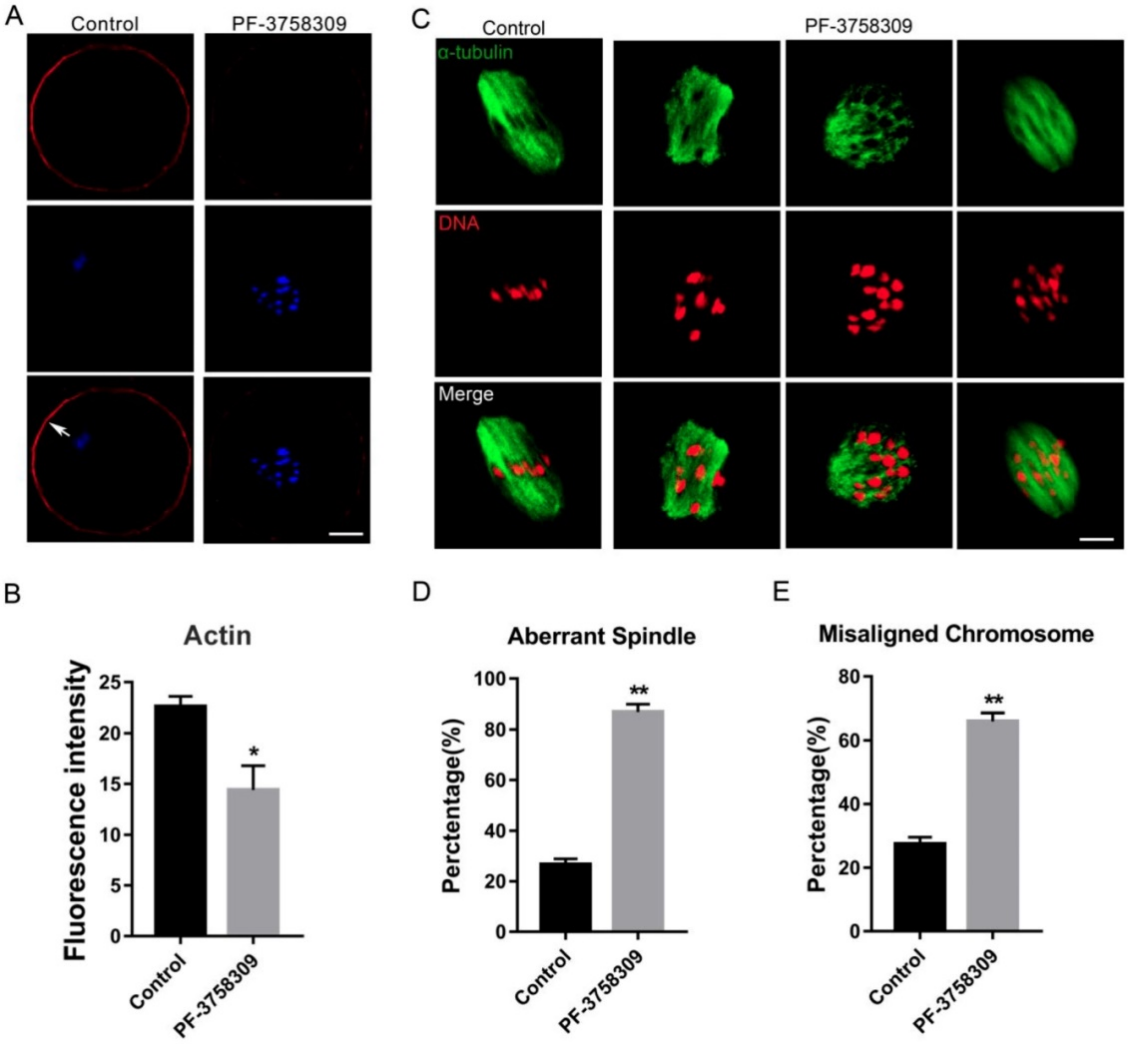
** PAK4 Inhibition results in the abnormal actin and microtubule assembly, accompanied with misaligned chromosomes during meiotic maturation of mouse oocytes. (A)** Representative images of actin distribution after PF-3758309 treatment in mouse oocytes. Actin (*red*) and DNA (*blue*). White arrow indicates actin cap. Bar = 25 μm. **(B)** Fluorescence intensity analysis of actin in both membrane and cytoplasm **(C)** Representative images of spindle morphology and chromosome alignment in the control and PF-3758309-treated oocytes. α-tubulin (*green*) and DNA (*red*) , Bar = 10 μm. **(D)** The rate of aberrant spindle formation in the control and PF-3758309 treatment groups. **(E)** The rate of misaligned chromosomes in the control and PF-3758309 treatment groups. Data are presented as mean ± SEM of at least three independent experiments. *P < 0.05, **P < 0.01.

To investigate whether PAK4 affects the assembly of actin and microtubule networks in meiosis, we cultured oocytes with 1 μM PF-3758309 for 8 hours, at which most oocytes developed to MI stage. Then oocytes were stained with fluorescent phalloidin for actin and anti-α-tubulin antibody for spindle morphology detection. The experimental results showed that actin signals on the oocyte membrane were significantly decreased after PAK4 inhibition by PF-3758309 treatment (Figure 3A), further approved by the fluorescence intensity analysis of actin signals (Figure 3B). The fluorescence intensities are 22.58 ± 1.02, n =84 (control) vs 14.4 ± 2.41, n = 91 (PF-3758309 group), P < 0.05. Also, in the PF-3758309-treated group, most of the spindles showed abnormal morphology and chromosomes misaligned in a broader pattern, compared with that the fusiform spindle and chromosomes with a regular linear pattern in the control group (Figure 3C). By the statistical analysis, the rate of abnormal spindle morphology and misaligned chromosomes were significantly increased in the PF-3758309-treated oocytes compared with that in the control oocytes. The rates of abnormal spindle morphology in the control and PF-3758309 groups are 26.58 ± 2.25%, n = 67 vs 86.77 ± 3.11%, n = 83, P < 0.01 (Figure 3D). The rates of misaligned chromosomes in the control and PF-3758309 groups are 27.4± 2.16%, n = 70 vs 65.97± 2.62, n = 69, P < 0.01 (Figure 3E). All these combined data demonstrated that PAK4 inhibition causes abnormal actin and microtubule dynamics, which further induces misaligned chromosomes during meiotic maturation progression.

### PAK4 inhibition leads to abnormal K-M attachment and aneuploidy during mouse oocyte meiosis

**Figure 4:**
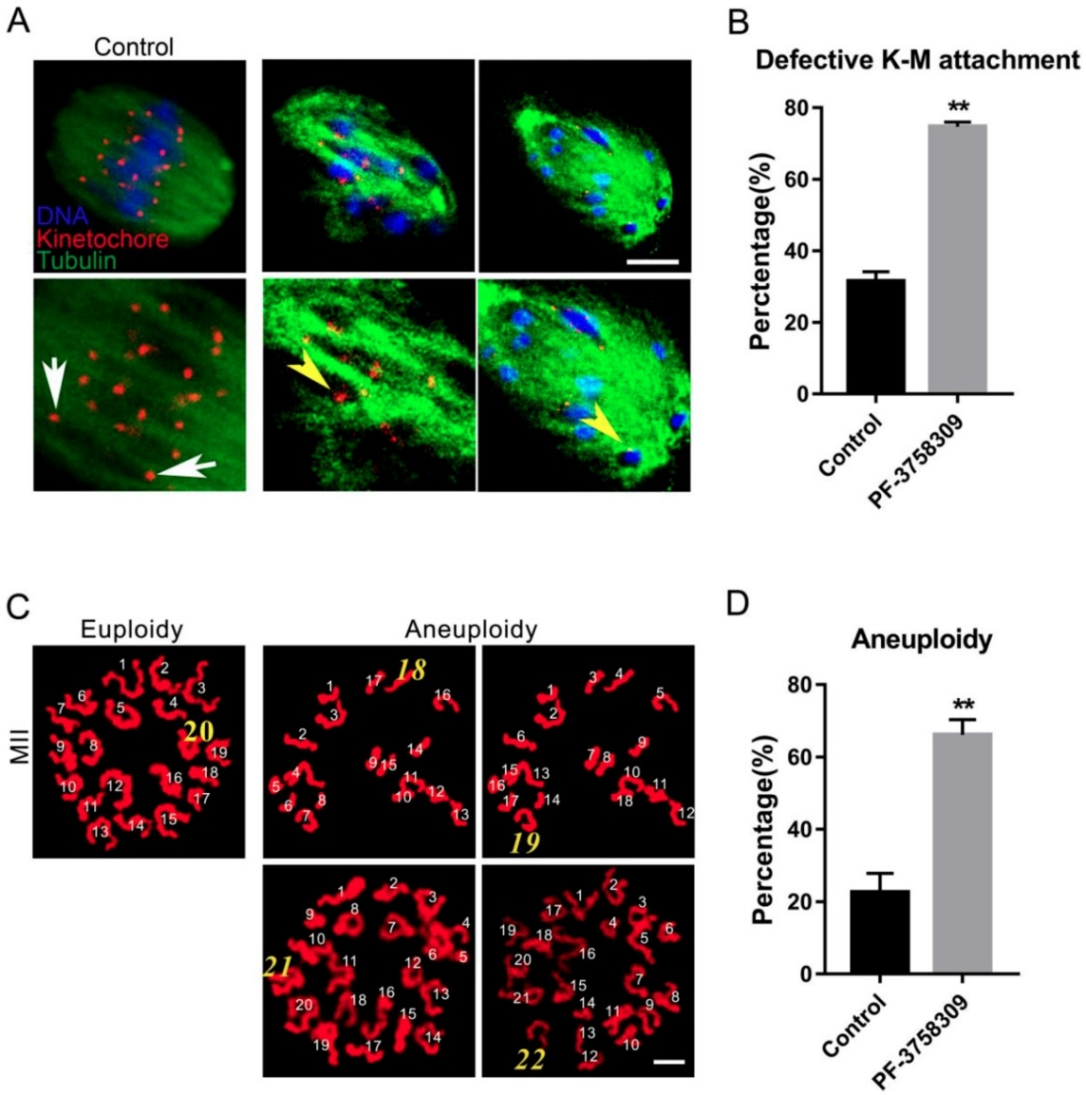
** PAK4 inhibition results in the defective kinetochore-microtubule (K-M) attachment and subsequent aneuploidy during meiotic maturation in mouse oocytes. (A)** Representative images of K-M attachment in the control and PF-3758309 treatment groups. Tubulin (*green*), kinetochore (*red*) and DNA (*blue*). White arrows indicate the normal K-M attachment and yellow arrowheads indicate the abnormal K-M attachment. Bar = 10 μm. **(B)** The rate of defective K-M attachment in the control and PF-3758309 treatment groups. **P < 0.01. **(C)** Representative images of euploidy (20 univalents) and aneuploidy (less or more than 20 univalents) in mouse oocytes with PBE. Bar = 5 μm. **(D)** The rate of aneuploidy in the control and PF-3758309 treatment groups. Data are presented as mean ± SEM of at least three independent experiments. **P < 0.01.

To further address the misaligned chromosomes caused by aberrant microtubule dynamics, we directly examined the K-M interaction by simultaneous kinetochore and microtubule staining as previously described, with anti-Centromere protein and anti-tubulin antibody, respectively 27. In most control oocytes, two kinetochores on one well-aligned chromosome were completely attached by microtubule fibers from two directions (Figure 4A, white arrows). However, in the PF-3758309-treated oocytes, dispersed kinetochores were not attached to microtubules from two directions, or only one kinetochore was attached to microtubule from one direction (Figure 4A, yellow arrowheads). The rate of aberrant K-M attachment in the PF-3758309-treated oocytes increased significantly compared to that in the control (Figures 4B). The rates of aberrant K-M attachment percentage in the control and PF-3758309 treatment groups are 31.43 ± 2.77%, n = 113 vs 74.7 ± 1.28%, n = 102, **P < 0.01. These defective K-M attachments will inevitably result in the establishment of unstable chromosome bi-orientation, which lead to unfaithful chromosome segregation. So we next analyzed the karyotype of mature oocytes with PBE by chromosome spread. As expected, in PF-3758309-treated oocytes, the chromosome numbers were more or less than 20, not as in the control (20 univalents, Figure 4C). The aneuploidy rate in the PF-3758309-treated group increased significantly compared with that in the control (Figure 4 D). The aneuploidy rates in the control and PF-3758309 treatment groups are 22.53 ± 3.05%, n = 57 vs 66.07 ± 2.47%, n = 61, **P < 0.01. These results indicate that PAK4 inhibition can lead to the defective K-M attachment which inevitably causes aneuploidy during meiotic maturation in mouse oocytes.

### PAK4 inhibition impairs the microtubule stability during mouse oocyte meiosis

**Figure 5:**
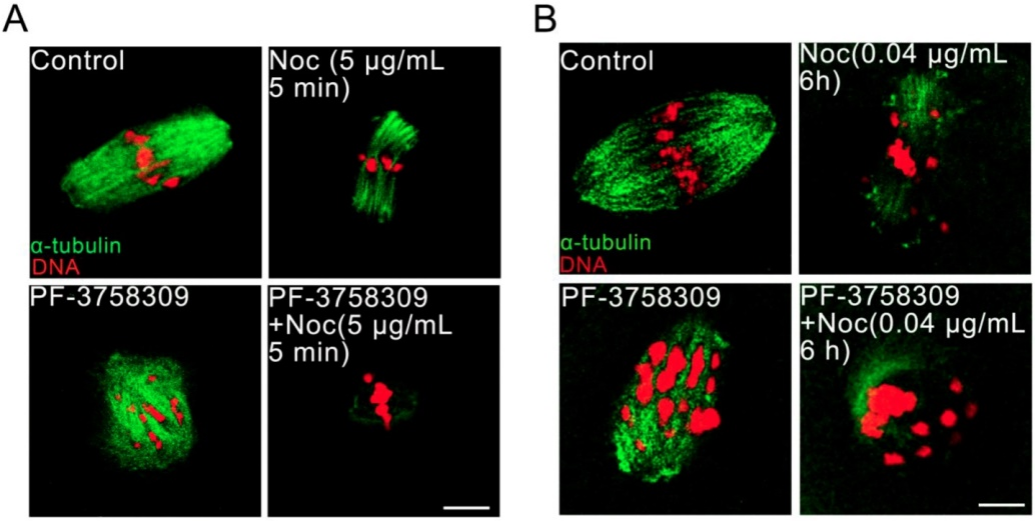
** PAK4 inhibition compromises the microtubule stability proved by microtubule depolymerization agent nocodazole during mouse oocyte meiosis. (A)** Representative images of microtubules in the control and PF-3758309-treated oocytes with 5 μg/mL nocodazole culture for 5 min. **(B)** Representative images of microtubules in the control and PF-3758309-treated oocytes with 0.04 μg/mL nocodazole culture for 6 h after GVBD. α-tubulin (*green*) and DNA (*red*). Bar = 10 μm.

**Figure 6:**
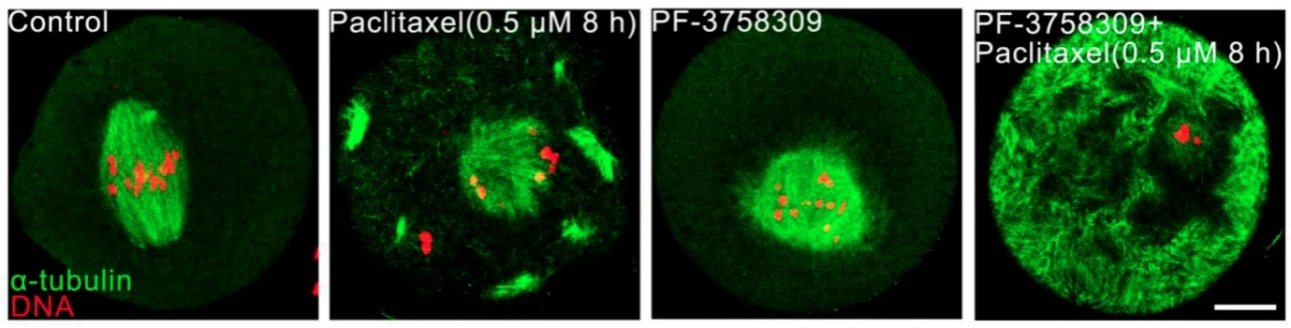
** PAK4 inhibition compromises the microtubule stability proved by microtubule polymerization stabilizer paclitaxel during mouse oocyte meiosis. (A)** Representative images of microtubules in the control and PF-3758309-treated oocytes with 0.5 μM paclitaxel culture for 8 h. α-tubulin (*green*) and DNA (*red*). Bar = 10 μm.

Aberrant spindles caused from PAK4 inhibition make us to further demonstrate the relationship between PAK4 and microtubule stability by using two oppositely functional microtubule drugs. One is microtubule depolymerization drug nocodazole and the other is microtubule polymerization stabilizer drug paclitaxel. In the control oocytes treated with nocodazole, microtubule fibers were still present, although the spindle morphology collapsed. However, microtubules were completely depolymerized in the PF-3758309-treated oocytes after the same treatment (Figure 5). Furthermore, in the paclitaxel group, the PF-3758309-treated oocytes exhibited snowflake-shape microtubule aggregation and the spindle morphology was completely destroyed (Figure 6). Thus, all these data from the two oppositely functional microtubule drugs indicate that PAK4 may regulate the microtubule stability during meiotic maturation in mouse oocytes.

### PAK4 inhibition induces acentriolar centrosome aberration during mouse oocyte meiosis

**Figure 7:**
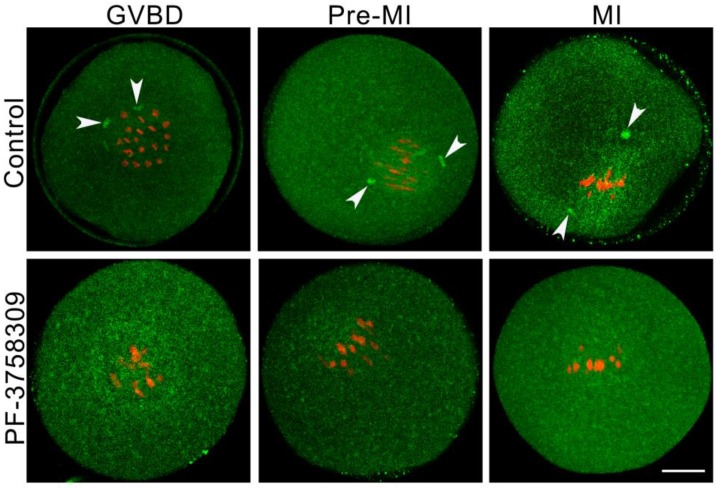
** PAK4 inhibition induces abnormal acentriolar centrosome assembly during mouse oocyte meiosis. (A)** Representative images of centrosomes at different development stages during mouse oocyte meiosis. Centrosome (*green*) and DNA (*red*). Bar = 20 μm.

The precise acentriolar centrosome assembly at meiotic spindle poles is important for the accurate microtubule cytoskeleton formation and faithful homologous chromosome segregation during oocyte meiosis 4. So we detected the centrosome assembly in the PF-3758309-treated oocytes with immunofluorescence by the anti-Pericentrin (centrosome marker) antibody. At each stage of meiosis I, the centromeric spots were present in the control group (white arrowheads), while absent in the PF-3758309-treated oocytes (Figure 7). These results confirmed that PAK4 may be necessary for the precise centrosome assembly at meiotic spindle poles in mouse oocytes.

## Discussion

PAK4 is the earliest discovered and the most representative member in group II PAKs which are mainly reported in mitotic cancer cell research. However, little is known about its functions during meiosis. In this study, we inhibited PAK4 functions by the highly selective inhibitor, PF-3758309, to investigate the PAK4 roles during meiotic maturation of mouse oocytes. Our data indicate that PAK4 inhibition causes meiotic arrest by impairing actin and microtubule dynamics during meiotic progression. PAK4 inhibition induces aberrant centrosome assembly, abnormal K-M attachment and subsequent aneuploidy. In conclusion, PAK4 is necessary for the oocyte meiosis as a regulator of cytoskeleton.

During meiotic maturation, oocytes undergo a series of consecutive events, such as GVBD, accurate spindle assembly, correct chromosome bi-orientation, faithful homologous chromosome segregation and PBE, then arrest at MII for fertilization. Cytoskeletons play important roles in cellular and molecular remodeling during meiotic maturation to perform functions in oocytes. PAK4 is required for the normal meiosis progression in oocytes because PF-3758309-induced PAK4 inhibition causes oocyte meiosis arrest. Before meiosis resumption and GVBD, the oocyte nucleus moves to the center from the periphery. This nucleus migration depends entirely on the actin network 32, 33. So abnormal actin dynamics caused by PAK4 inhibition could delay the actin-dependent mechanism to center the nucleus and the subsequent GVBD. Oocyte failure to extrude the first polar body is also due to the formation of microfilaments was blocked. As a cytoskeleton component, microfilaments are involved in many events in oocyte meiosis maturation, including GVBD, spindle assembly, chromosome alignment at the equator plate and PBE 32. At MI stage of oocyte, microfilaments were enriched in the cortical region to form an "actin cap" 32. During the meiotic maturation, spindle is pulled by the microfilament and moves from the central region to the cortical region of oocyte. Finally, oocyte undergoes the highly asymmetric splitting to extrude a polar body with a small volume. In this study, no actin cap was detected and the fluorescence intensity of actin was significantly weakened in PAK4-inhibited oocytes. So PAK4 inhibition may impair the microfilament dynamics, which eventually leads to the meiosis arrest. In mammalian oocytes, the spindle begins to form when homologous chromosome association occurs. Microtubule-dependent cortical pull is a major factor which continuously regulates the correct spindle localization 34. Oocyte meiosis maturation is closely related to the accurate assembly of spindle, so we next examined the spindle morphology at MI stage. Our data showed that PAK4 inhibition induces aberrant spindle formation and misaligned homologous chromosomes. Therefore, PAK4 inhibition may disrupt the interaction among microtubules, thereby preventing the stable fusiform-shaped spindle establishment and correct homologous chromosome alignment.

The abnormal spindle morphology also reminds us that the correct K-T attachment may be impaired simultaneously. In meiosis I, faithful homologous chromosome segregation requires the kinetochores attachment to the microtubules from the opposite spindle poles, termed as bi-orientation contrast to mono-orientation in mitosis 3. Defects of bipolar K-M attachment inevitably lead to oocyte aneuploidy 35. It is estimated that 15-20% of oocytes undergo unfaithful chromosome segregation 36 and that 5% of pregnancies suffer aneuploidy in human 37. In this study, we observed a significantly higher rate of defective K-M attachment in the PAK4-inhibited oocytes compared with the control. The oocytes with defective K-M attachment in MI are particularly susceptible to non-segregation, leading to aneuploidy. Mouse has 40 telocentric chromosomes, so homologous chromosomes form 20 bivalents (cross shape and kinetochore at the end) in MI, while chromosome spread is usually conducted in MII oocyte with 20 univalents 28, 38. Our next chromosome spread assay further proved the aneuploidy from the defective K-T attachment in the PAK4-inhibited oocytes. Oocyte euploidy is critical for early embryo development and the genome integrity. So even these inhibitor-treated oocytes were fertilized successfully, they could not develop normally as expected. We hypothesized that PAK4 inhibition may generate less force generated by the microtubules, which mechanically exerts less pull tension on the kinetochores of homologous chromosomes, thereby resulting in the disorder of K-T attachment and eventually producing aneuploidy.

All the microtubule defects from PAK4 inhibition promote us to further investigate the PAK4 participation in the meiotic microtubule stability. We treated the PAK4-inhibited oocytes with two opposite microtubule drugs: nocodazole is a microtubule depolymerization drug, while paclitaxel promotes microtubule polymerization. We found that PAK4 inhibition wakens the microtubule stability resistant to nocodazole. Notably, paclitaxel can promote tubulin polymerization and maintain tubulin stability. However, PAK4 inhibition still reduces the microtubule stability even with paclitaxel treatment. This microtubule stability may be related to the phosphorylation of tubulin since PAK4 is a kinase 39. In conclusion, both the anti-microtubule drug experiments suggest that PAK4 plays an important role in the regulation of microtubule stability.

**Figure 8:**
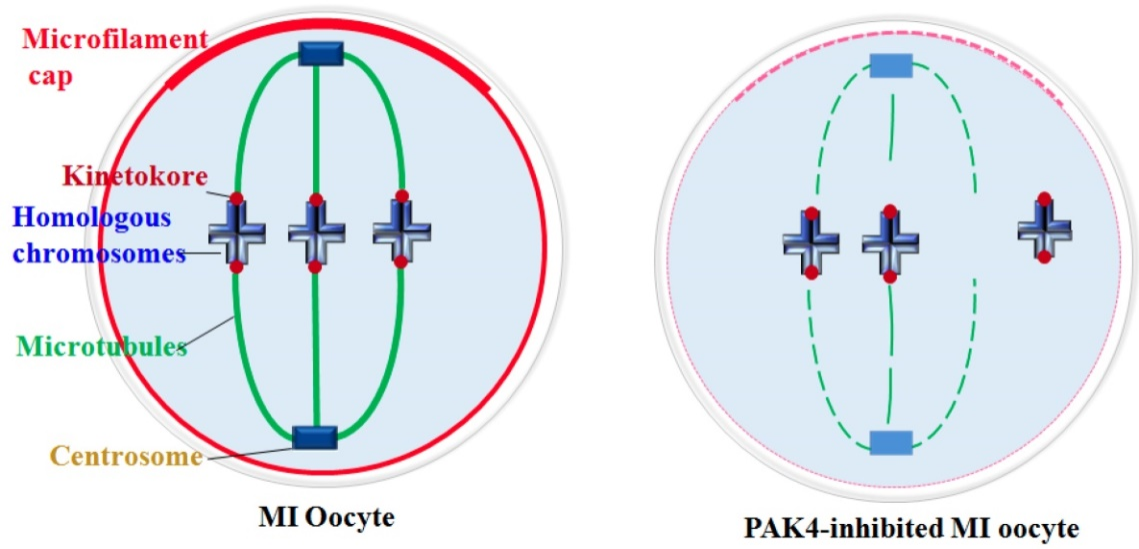
Schematic diagram showing PAK4 inhibition affects the microfilament and microtubule dynamics during mouse oocyte meiosis.

Centrosome plays essential functions in the accurate spindle formation and symmetric division in mitotic cells. While during meiosis in germ cells, atypical centrosome without centrioles performs the similar functions to the mitotic centrosome with centrioles 4. So some people insist to use the term of microtubule organizing center (MTOC) or acentriolar centrosomes instead of centrosome in meiosis. In despite of the dispute, it is clear that the centrosomal material complex around the spindle poles is the most important region for the microtubule initiation and accurate spindle assembly in both meiosis and mitosis. Then spindle microtubules with other factors pull chromosomes to the opposite cell poles during every cell cycle, so each daughter cell presents a complete set of genome^40^. In this experiment, we detected the centrosome formation and found that centrosome assembly is completely impaired in the PAK4-inhibited oocytes during meiotic maturation. So PAK4 is required for the meiotic maturation as a regulator of cytoskeletal integrality through connection with centrosome assembly. A schematic diagram of PAK4 inhibition inducing meiotic cytoskeleton errors is shown in Figure 8.

Cytoskeletal remodeling is an important event during oocyte meiotic maturation. Oocytes undergo significant changes in microfilament and microtubule dynamics and centrosome assembly to form the accurate meiotic spindles. Our data suggest that PAK4 is essential for the meiotic maturation in mouse oocyte as a regulator of cytoskeletal integrality through connection with microfilaments, microtubules, K-M attachment and centrosome assembly.
